# Trust the crowd: Crowdsourced fact-checking is as effective at reducing confidence in misinformation as expert fact-checking

**DOI:** 10.1371/journal.pone.0348291

**Published:** 2026-05-20

**Authors:** Cindy Phan Vu, Lauren L. Saling

**Affiliations:** School of Health and Biomedical Sciences, RMIT University, Melbourne, Australia; University of Science and Technology of Fujairah, YEMEN

## Abstract

The rapid spread of misinformation on social media has created significant challenges for expert fact-checking initiatives to counter in a timely and effective manner. Misinformation undermines behaviour and decision-making in many spheres including health and political domains. X (formerly known as ‘Twitter’) utilises crowdsourced fact-checking (termed ‘Community Notes’) to manage the high volume of and engagement with online misinformation. Community Notes have also been introduced to mitigate perceived partisanship and bias of expert fact-checkers. The present study recruited 102 participants to investigate whether expert or crowdsourced fact-checks on X are more effective at reducing belief in misinformation and engagement with misinformation. Participants were randomly allocated into either an expert or crowdsourced fact-checking condition. Confidence in the veracity of misinformation and willingness to retweet were measured, before and after exposure to fact-checks. It was found that both crowdsourced and expert fact-checks reduced confidence in misinformation and willingness to retweet the information. The results demonstrate the efficacy of crowdsourced fact-checking, a fact-checking variant that is rapidly gaining popularity. Given this, the adoption of crowdsourced fact-checking by other social media platforms warrants consideration.

Social media has become a mechanism for the rapid dissemination of both information and misinformation [1]. Misinformation refers to inaccurate, uncontextualized, or false information, irrespective of whether it is spread to misinform others [2]. Against the backdrop of the COVID-19 pandemic and recent U.S. elections, there has been an emphasis on counteracting misinformation through fact-checking mechanisms [3]. Misinformation undermines behaviour and decision-making, especially within political and health-related domains [4,5]. During the COVID-19 pandemic, belief in the veracity of misinformation on social media influenced individuals to reject measures to curtail the spread and severity of the virus including social distancing and vaccines [6]. Hence, social media platforms have made efforts to mitigate its spread as well as inform individuals about accurate information through fact-checking initiatives.

There are different sources of fact-checks, for instance, expert, AI and crowdsourced [7]. AI fact-checking refers to the use of automated systems and large language models to detect and label potentially false or misleading content, typically by cross-referencing claims against verified information sources or flagging content for human review [8]. While AI fact-checking offers the potential for speed and scale that neither expert nor crowdsourced approaches can match, concerns remain about accuracy, hallucination, and the absence of human judgement in evaluating nuanced or contextually sensitive claims. The present study focuses on expert and crowdsourced fact-checking, as these represent the two most widely deployed human-driven approaches on X and the comparison most directly relevant to current platform policy decisions.

Independent expert fact-checkers (e.g., FactCheck.org, PolitiFact, Snopes) employ objective, evidence-based methods to evaluate claim veracity, drawing on reliable primary sources and individuals with relevant expertise [9]. Expert fact-checkers prioritise objectivity, but acknowledge that information can be interpreted differently by individuals with different political affiliations [9]. Studies have shown that expert debunking can reduce misperceptions about misinformation veracity, demonstrated for claims made by politicians [10], health [7], or vaccines [11].

However, people do not always trust expert fact-checkers (Brandtzaeg et al., 2018). A mixed-methods study [12] analysed social media conversations to assess attitudes towards fact-check organisations. Social media users expressed some ambivalence towards fact-check organisations, particularly with respect to objectivity. One source of scepticism is a fear that partisanship may influence what information is fact-checked [10]. Hence, individuals may reject or ignore debunking, if they assume that fact-checkers are pushing a particular political agenda [13]. While expert fact-checking has a demonstrated ability to reduce trust in misinformation, lack of public trust in fact-checkers arising from perceived bias or partisanship has encouraged alternative sources of fact-checks. Furthermore, since misinformation spreads rapidly on social media, expert fact-checking is not always effective within an appropriate timeframe [13]. When posts containing misinformation persist without fact-checks, users may assume that the post is true [14]. Thus, investing in efficacious and timely alternative fact-checking mechanisms may prove valuable.

Crowdsourced fact-checking has been proposed as a timely and less partisan alternative to expert fact-checking [15]. However, note that crowdsourced fact-checkers have been found to more commonly fact-check claims from those with opposing views potentially reflecting a partisan bias [16]. X introduced Community Notes (formerly Birdwatch, launched in January 2021 and rebranded in November 2022) to tackle misinformation through crowdsourced fact-checking [17]. The model has since attracted considerable uptake across the social media landscape. YouTube piloted a comparable system in June 2024, Meta announced the replacement of its expert fact-checking program with Community Notes across Facebook, Instagram, and Threads in January 2025 [18], and TikTok launched its own iteration — “Footnotes” — in April 2025. The increasing adoption of this approach across major platforms underscores the importance of evaluating whether crowdsourced fact-checking is effective and whether it should be deployed more broadly, or whether resources would be better directed toward alternative fact-checking mechanisms.

Community Notes involves laypeople identifying X posts with misinformation and creating a note containing a fact-check. These appear on the post when a diverse group of X users have agreed that the fact-check was helpful. Research has highlighted that belief in the accuracy of crowdsourced fact-checks tends to occur when there is collective agreement among users [19]. This effect was found for claims labelled as true by an AI or crowdsourced fact-check but not for claims labelled as false. However, the study only compared crowdsourced fact-checks with AI fact-checks and not expert fact-checks. Additionally, the fact-checks only consisted of labelling claims as true or false but did not provide corrective information. Thus, comparing crowdsourced fact-checks with expert fact-checks, and examining the role of corrective information, could help determine which mechanism is more effective in reducing belief in the veracity of misinformation.

As crowdsourced fact-checking relies on laypeople’s judgements, it is important to determine whether crowdsourced fact-checkers can produce high-quality fact-checks. The fact-checking process involves both the identification of misinformation [20] and the retrieval of high-quality evidence to counter the misinformation [21]. Unlike expert fact-checkers, there are no guidelines for crowdsourced fact-checkers. However, studies have revealed a high level of agreement between expert and community fact-checks [21–23] despite some important differences. For example, the variety of claims targeted by crowdsourced fact-checkers have been claimed to be more expansive [23] than those investigated by expert fact-checkers [9]. The fact-checks produced by laypeople may be done in an overconfident manner or be impacted by cognitive biases [24]. Thus, complete reliance on crowdsourced fact-checks may be problematic, and seeking convergence with expert fact-checks could prove beneficial when attempting to mitigate the impact of misinformation. Interestingly, a report prepared by Fundación_Malditas [25], a fact-checking organisation based in Europe, has found that X Community Notes that include a link to a fact-checking organisation are more trusted by users, are posted more quickly and are more likely to be posted than those without this feature. This inter-dependence between expert and community-based fact-checks suggests that drawing on both mechanisms may prove more effective when attempting to mitigate the impact of misinformation.

Due to the relative novelty of crowd-sourced fact-checking, there is a dearth of literature concerning its efficacy in influencing updating of veracity judgements. Most of the extant literature regarding crowdsourced fact-checking on social media analysed X data and investigated Community Notes’ performance in terms of rate of misinformation identification and engagement [26,27]. Crowdsourced fact-checkers on average identified and debunked misinformation at a faster rate than expert fact-checkers [27]. Yet even though the community is effective in detecting misinformation, crowdsourced fact-checkers are still unable to correct posts before virality, so misinformation has already reached a wide audience. Indeed, Chuai, Tian [26] found that crowdsourced fact-checking did not reduce engagement (liking or re-sharing a post) with misinformation. Although this suggests that crowdsourced fact-checks are not as effective as they could be, likes and shares are not the only indication of fact-check efficacy, as users may engage with posts they don’t judge to be accurate [28].

A further consideration bearing on the interpretation of fact-checking research concerns the broader information environment within which fact-checking operates. Zhou, Yang [29] employed observational panel data tracking the web browsing behaviour of approximately 140,000 individuals in the US over twelve months. The authors demonstrate that exposure to misinformation is not evenly distributed across users but is concentrated among those who are more informed and more heavily engaged with news. Importantly, their analysis shows that the top 1% of panellists were responsible for visiting 65.3% of all unreliable pages — a distribution far more skewed than for reliable news content. This “heavy user” pattern suggests that both misinformation and its potential corrections disproportionately reach a narrow subset of highly active users rather than the general population.

Complementing this at the level of fact-checking specifically, Bhalla, Ray [30] analysed data from over 7 million Twitter/X users across the United States and India finding that exposure to and engagement with fact-checks — whether measured by following, retweeting, or replying — remains largely restricted to the heaviest users, with little evidence that these interventions penetrate among selectively partisan news audiences. Importantly, they found that following partisan outlets was associated with reduced odds of following fact-checkers, irrespective of partisan direction. The broader information environment within which users encounter both misinformation and its corrections also matters: Bhalla, Ray [30] argue that in high-choice media environments driven by commercial and entertainment logics, corrective information operates at a structural disadvantage, with audiences’ responses to fact-checks shaped by the same partisan frameworks that the information environment itself has constructed. Taken together, these findings suggest that the key bottleneck in real-world fact-checking effectiveness may be less the credibility or source of the fact-check than the degree to which fact-checking reaches users in naturalistic settings at all. The present study addresses the question of fact-check efficacy under conditions of guaranteed exposure, and its findings should be interpreted with this constraint in mind.

## The present study

Despite efforts by social media platforms, a lack of timely posting and perceived partisanship and bias of expert fact-checkers can reduce the effectiveness of fact-checking initiatives. With the introduction of Community Notes by X, and subsequently by other platforms, it is important to investigate whether crowdsourced fact-checking is effective and therefore should be more widely deployed across social media platforms or if efforts should be directed more towards expert fact-checks. Thus, the aim of the present study was to investigate which fact-checking source, expert or crowdsourced, is more effective in reducing confidence in misinformation.

The study employed two categories of claims: false claims, which contained misinformation about homelessness, and true claims, which accurately represented the state of homelessness. This distinction is theoretically and methodologically important for several reasons. First, it allowed us to assess the differential effects of fact-checking on beliefs that participants held with varying degrees of prior confidence. Participants may be more confident in the veracity of true claims than false claims prior to exposure to any fact-checks, thus claims may differ meaningfully in their initial perceived plausibility. This raises the question: do fact-checks operate differently depending on whether they confirm or disconfirm a person’s pre-existing credence in a claim?

For false claims, fact-checks may provide corrective information that challenges an existing belief of moderate confidence. For true claims, it may be the case that fact-checks instead serve a confirmatory function, potentially reinforcing beliefs that participants already held with relatively high confidence. These are conceptually distinct epistemic processes — correction versus confirmation — and both are relevant to understanding how fact-checking mechanisms function in practice. A comprehensive account of fact-checking efficacy should encompass not only whether fact-checks successfully reduce confidence in misinformation, but also whether they strengthen correctly held beliefs in an information environment where the veracity of claims is often uncertain. Accordingly, analyses were conducted separately for false and true claims to allow assessment of both functions.

Given that expert fact-checks are better established than crowdsourced fact-checks and that crowdsourced fact-checks are considered more accurate when they converge with expert fact-checks, the following hypotheses were tested:

H1. X users exposed to posts by an expert fact-checker will have a higher reduction in confidence in the veracity of misinformation than those exposed to Community Notes.
H2. X users exposed to posts by an expert fact-checker will be less inclined to share the post containing misinformation than those exposed to Community Notes.

It is worth noting that the present study, by design, ensures participant exposure to fact-checks — a condition that does not hold in naturalistic social media environments. Ray, Bhalla [31] demonstrate that in real-world settings on X, engagement with fact-checking content is extremely rare and concentrated among a small subset of highly active users. The present findings therefore speak to the relative efficacy of crowdsourced versus expert fact-checks under conditions of controlled exposure, rather than to their comparative real-world reach or population-level impact.

## Method

This study, including hypotheses and analysis plan, was pre-registered at OSF https://osf.io/4vz6j/?view_only=d53f10c55ed446c28b109c837cf9b685

### Participants

An a priori power analysis, conducted using G*Power 3.1 [32] for a 2x2 mixed model analysis of variance (ANOVA) with power = .8, and effect size = .2, revealed that a minimum of 52 participants was required. Inclusion criteria were that individuals (i) have used X at least once a month and have tweeted 20–100 times in the past 12 months, (ii) were at least 18 years of age, (iii) were proficient in English and (iv) reside in Australia, United Kingdom, or the United States. The final sample consisted of 102 participants with an age range 18–68 years (*M* = 39.2, *SD* = 12.0). Table 1 presents sample characteristics as a function of condition. Political ideology was categorised on a 100-point scale with Liberal = 0–40 and Conservative = 60–100. For liberals, M = 18.31, SD = 13.41; for conservatives, M = 74.52, SD = 14.34 and for the full sample; M = 39.12, SD = 28.17. Participants who nominated a point on the slider between 41 and 59 were classified as neutral. All participants who were classified as neutral selected 50 on the slider.

**Table 1:** Participants’ Demographics for Expert and Crowdsourced Fact-Check Conditions.

	Expert		Crowdsourced		
Demographics	n	%	n	%	Total
*Gender*					
Male	35	34.3	34	33.3	69
Female	16	15.7	15	14.7	31
Non-binary/Other	0	0	2	2.0	2
*Education level*					
High School	15	14.7	17	16.7	32
Post high-school diploma	5	4.9	11	10.8	16
Bachelor’s degree	20	19.6	17	16.7	37
Postgraduate degree	11	10.8	6	5.9	17
*Political Ideology*					
Liberal	28	27.5	31	30.4	59
Conservative	17	16.6	14	13.8	31
Neutral	6	5.9	6	5.9	12

Total n = 120.

### Measures and materials

#### Demographics.

Non-identifying demographic measures: age, gender, political ideology and highest level of education completed, were collected.

#### True and false claims.

Two true and four false claims were used for this study and were constructed to comply with the format of X/Twitter posts. We adopted a similar ratio to that used by Carnahan and Bergan [33] who used a 1:4 ratio of true to false claims. The rationale for this ratio is that it approximates the mix of claims encountered in a real-world information environment and because the focus of our study was on whether people judge misinformation to be true rather than their judgements of true claims per se. Claims were created using an X post generator [34]. Homelessness was selected as a topic as it is not overly politically charged and a topic with which participants were likely to be less familiar [35]. Claims were created from information provided by homelessness organisations and government bodies [35–38]. Engagement numbers (likes, retweets, and views) were made to resemble viral X posts. Claims are presented as text only in Supplementary materials (S1 File).

After viewing each claim, participants were asked to (i) indicate their confidence in the statement, on a response scale from 0–100% likely to be true, and (ii) likelihood of retweeting the post on a response scale from 0–100%.

#### Fact-checks.

Crowdsourced fact-checks were created to resemble Community Notes and expert fact-checks were created to resemble real X posts by an expert fact-checker. Community Notes were created using a Community Notes generator [39]. Like the claims, expert fact-checks were created with an X post generator [34]. Community-notes and expert fact-checks are presented as text only in supplementary materials (S1 File). The chosen source of expert fact-checks was Reuters Fact-Check, as this has been rated as reliable and politically neutral [40]. Engagement numbers were made to resemble the actual engagement received by the Reuters Fact-Check X account.

### Procedure

This study was approved by RMIT University’s Human Research Ethics Committee (project no: 27606). The study complied with ethical standards of the Declaration of Helsinki. Consent was provided through completion of a consent question and implied through submission of study responses. No identifying information was collected from participants.

Data were collected from 23/08/2024-24/08/2024. Participants were recruited through Prolific, an online recruitment platform, and remunerated with $2.94 AUD (approximately $1.84 USD). Median completion time was 6.13 minutes. The anonymous online study was completed via Qualtrics. After completing the demographics questions, participants were randomly allocated to either the crowdsourced fact-check condition or the expert fact-check condition using the Qualtrics randomiser function. They were then shown the six claims about homelessness, with the order of presentation randomised. Note that the X posts and associated expert fact-checks and Community Notes were created for this study by the researchers and were therefore not sourced from X or any third-party dataset.

After reading each claim, participants responded to the two questions: likelihood to be true and willingness to retweet (both measured on scales ranging from 0–100%). Participants were then shown the relevant fact-checks, either Community Notes or Reuters Fact Checks corresponding to each claim, depending on their group allocation. Fact-checks were shown alongside the original claim. After exposure to each fact-check, participants again responded to the two questions about each claim. After study completion, participants were provided with a debriefing statement and a fact sheet about homelessness [41].

## Results

Statistical analyses were conducted using Jamovi [42]. Table 2 presents descriptive statistics for the two questions for false and true claims separately.

**Table 2:** Descriptive Statistics for the two questions.

Claim Type	Likely to be true		Willingness to Retweet	
	Pre-Fact-Check *M (SD)*	Post-Fact-Check *M (SD)*	Pre-Fact-Check *M (SD)*	Post-Fact-Check *M (SD)*
False Claims				
Expert	55.4 (12.5)	49.2 (18.2)	26.2 (19.3)	25.3 (19.6)
Crowdsourced	55.0 (14.0)	47.4 (20.7)	28.7 (23.7)	26.3 (22.7)
True Claims				
Expert	75.2 (17.9)	77.4 (19.7)	73.1 (18.1)	46.8 (33.8)
Crowdsourced	73.1 (18.1)	80.5 (18.0)	42.4 (29.4)	52.7 (33.3)

Note: For both questions: likely to be true and willingness to retweet, the response scale was a slider ranging from 0–100%.

Assumptions were checked prior to undertaking analyses. Levene’s test revealed no violation of homogeneity. Inspection of Q-Q plots and histograms revealed mild violations of normality but given that ANOVAs are generally robust against normality violations no transformations were conducted. Participants’ belief in misinformation and willingness to retweet were averaged across claims. Analyses were conducted for false and true claims separately.

To test hypotheses 1 and 2, a series of 2x2 mixed model ANOVAs were performed. For all analyses, the within-subjects variable was time (pre- and post-fact-check), and the between-subjects variable was condition (expert and crowdsourced). For H1 the dependent variable (DV) was confidence in the likelihood of claim veracity. For H2, the DV was willingness to retweet.

### Confidence in the likelihood of claim veracity (Hypothesis 1)

#### False claims.

There was a significant main effect of time with confidence in likelihood of being true significantly reducing post-fact-check, *F*(1,100) = 22.27, *p* < .001, η_p_^2^ = 0.182, indicating a large effect size. There was no significant main effect of condition, *F*(1,100) = 0.51, *p* = .698, and the interaction was not significant, *F*(1,100) = 0.23, *p* = .630.

#### True claims.

There was a significant main effect of time, *F*(1,100) = 9.46, *p* = .003, η_p_^2^ = 0.086 (medium effect size) with an increase in confidence in claim veracity post-fact-check. The main effect of condition, *F*(1,100) = 0.03, *p* = .871 and the interaction between condition and time were non-significant, *F*(1,100) = 2.82, *p* = .096.

### Willingness to retweet (Hypothesis 2)

#### False claims.

The main effect of time *F*(1,100) = 2.27, *p* = .135, condition, *F*(1,100) = 0.18, *p* = .676, and the interaction, *F*(1,100) = 0.54, *p* = .479, were non-significant.

#### True claims.

There was a significant main effect of time with an increased willingness to retweet post-fact-check, *F*(1,100) = 26.98, *p* < .001, η_p_^2^ = 0.212. There was no significant main effect of condition, *F*(1,100) = 0.24, *p* = .628. A significant interaction emerged between time and condition, *F*(1,100) = 4.61, *p* = .034, with a small effect size, η_p_^2^ = 0.044 explained by an increase in willingness to retweet following exposure to the fact-check in the crowdsourced condition.

## Discussion

The current study investigated changes in confidence in misinformation after exposure to either expert or crowdsourced fact-checks on X. Hypothesis 1 (“X users who were exposed to an expert fact-checker would have a higher reduction in confidence in belief in misinformation than those exposed to Community Notes”), was not supported. With extant literature demonstrating the effectiveness of expert fact-checking as a mechanism to tackle misinformation [7,11] it was expected that this fact-check source would be more effective than Community Notes. Although we found that for false claims, for both the expert and community fact-check groups there was a reduction in confidence in belief in misinformation, there was no significant difference in the amount of reduction as a function of fact-check source. Indeed, we found a slightly higher mean change in the community fact-check group. For true claims, for both expert and community fact-check groups, there was an increase in confidence of belief in misinformation, however, there was no significant difference in the amount of change as a function of fact-check type.

Hypothesis 2 (“X users who were exposed to an expert fact-check would be less inclined to share the post containing misinformation than those exposed to Community Notes”), was also not supported. For false claims, willingness to retweet did not change significantly post fact-check for either condition. Interestingly, for true claims, those in the expert fact-check group were less willing to re-tweet after exposure to the fact-check, while those in the community fact-check group were more willing to re-tweet.

Our findings align with those of previous studies, where fact-checking has been demonstrated to be effective in reducing belief in misinformation [10,43]. The reduction in confidence in information veracity in both expert and community fact-check conditions suggests that fact-checking (irrespective of the source) can provide alternative evidence-based information that users are willing to accept. In the context of social media, implementing fact-checks from a range of sources is likely to assist in reducing confidence in misinformation veracity and challenging false beliefs [33]. In the current study, exposure to fact-checks also enabled participants to confirm their existing beliefs in true claims, as confidence in the veracity of these claims (and willingness to retweet for the Community Notes group) increased following exposure to fact-checks. The strengthening of correctly held beliefs may be particularly valuable in the contemporary information landscape, where individuals are routinely exposed to information of uncertain veracity.

These findings warrant interpretive caution regarding what the observed equivalence between crowdsourced and expert fact-checks means in practice. While the conditions produced comparable reductions in confidence in false claims, this equivalence under controlled-exposure conditions may not translate into equivalent real-world impact. Ray, Bhalla [31] show that engagement with fact-checks in naturalistic settings is heavily concentrated among the platform’s heaviest users — those who follow a disproportionately large number of sources and post and like at higher rates than typical users. The implication is that the more fundamental constraint on fact-checking effectiveness may not be source credibility but reach: most users may never encounter either type of fact-check. A more precise characterisation of the present findings is therefore that both crowdsourced and expert fact-checks can reduce confidence in misinformation when users see them — a meaningful finding, but one that leaves open the question of real-world population-level impact.

The finding that neither condition significantly reduced willingness to retweet false claims, and that the crowdsourced condition increased willingness to retweet true claims, should be interpreted given broader platform dynamics. Bhalla, Ray [30] argue that in high-choice media environments structured around entertainment logics, audience responses to corrective information are shaped less by the content of the correction than by partisan and cultural frameworks the broader information environment has already constructed. These patterns suggest that fact-checks may function partly as engagement signals rather than purely as corrective interventions, potentially amplifying as well as correcting content, in ways that only a richer account of the surrounding information environment can fully explain.

The efficacy of Community Notes in correcting misinformation and (ideally) associated underlying false beliefs as well as affirming the accuracy of true information could be attributed to the power of collective agreement and peer endorsement [25]. A community of X users aggregating to contribute to a fact-check may elicit a similar effect to that observed with peer endorsement in consumer-related advertisements [44]. However, it is worth subjecting the collective intelligence logic that implicitly underpins Community Notes to scrutiny. The classic formulation of the ‘wisdom of crowds’, most associated with Surowiecki [45], holds that accurate aggregate judgements require independence of individual opinions, diversity of perspectives, and decentralised participation representative of the broader population. Community Notes may not fully satisfy these conditions. As Ray, Bhalla [31] demonstrate, participation in fact-checking activities on X is disproportionately concentrated among heavy users who consume a broad range of sources and follow partisan outlets on multiple sides, a group not representative of the general user population. Zhou, Yang [29] similarly show that the users most likely to encounter unreliable content are those with the most extensive and diverse news diets, suggesting that Community Notes contributors may share more in common than a genuinely representative crowd would. Rather than harnessing distributed epistemic diversity, Community Notes may therefore reflect the views of a narrow, highly engaged subset of users. This does not negate the findings reported here but suggests that the mechanism underlying Community Notes’ effectiveness may be better understood in terms of perceived peer endorsement rather than as a genuine ‘wisdom of the crowd’ effect.

Including Community Notes as a fact-check mechanism in combination with fact-checks from other sources (including expert and AI fact-checks) is likely to offer more timely and efficacious correction of misinformation than expert fact-checks alone. AI-assisted fact-checking represents an increasingly prominent third approach that was beyond the scope of the current study. However, X has recently piloted AI-generated Community Notes, where large language models contribute notes that then undergo the same human peer-review process as contributor-authored notes. Future research comparing the efficacy of AI, crowdsourced, and expert fact-checks — both independently and in combination — would substantially advance understanding of how best to deploy these mechanisms.

## Limitations and future research directions

This study had some limitations which require consideration. Retweeting was used as a measure of sharing of and engagement with the information. However, for some users retweeting is not the mechanism demonstrating engagement [46]. Indeed, some participants in the present study rated their willingness as “0%” for all the claims, pre- and post-fact-check. Alternative measures of engagement or sharing such as liking a post or commenting, could be used in future studies. An interesting consideration for future studies could also be to incorporate a longitudinal element. Carnahan and Bergan [33] indicated that the effects of fact-checking were rather short-lived and therefore it would be of value to determine whether the effects seen here persist over time and generalise to other content.

It should also be noted that the current study’s generalisability is constrained by several features of the design. The stimulus materials comprised only six posts (four false, two true) drawn from a single platform, addressing a single topic, and representing expert fact-checking through a single source. It therefore remains an open empirical question whether the observed effects would replicate across a broader range of topics, platforms, and fact-checking implementations. Future research could usefully extend the current findings by examining crowdsourced and expert fact-checks across multiple content domains and social media platforms, and by incorporating emerging fact-checking modalities including AI-assisted fact-checking.

## Implications and conclusion

This is one of the first studies to investigate the efficacy of Community Notes in reducing belief in misinformation and to compare the efficacy of this fact-checking mechanism to expert fact-checks. Our findings suggest that crowdsourced fact-checks can be as effective as expert fact-checks to reduce people’s confidence in the veracity of misinformation. An implication is that social media platforms could employ fact-checks from a variety of sources to manage the pace and volume of misinformation. Crowdsourced fact-checking should not replace expert fact-checking, rather these approaches are complementary. The goal is to ensure that posts containing misinformation on social media rapidly receive some form of fact-check to lessen the likelihood that they are perceived as true.

As misinformation continues to proliferate on social media, it is imperative to investigate multiple sources types of fact-checks to effectively combat its reach. Behaviours executed based on belief in the veracity of misinformation can be inherently dangerous or harmful, as observed during the COVID-19 pandemic [6]. Whilst fact-checking is not a stand-alone mechanism for shifting false beliefs and associated behaviours, it provides the opportunity for individuals to consider alternative, accurate information.

## Supporting information

S1 File False and True Claims, Community Notes and Expert Fact-Checks created for this study. (DOCX)
