# Supplementary material for: Trust the crowd: Crowdsourced fact-checking is as effective at reducing confidence in misinformation as expert fact-checking
Source: PLoS One. 2026 May 20;21(5):e0348291. doi: 10.1371/journal.pone.0348291 (PMC13189315; doi:10.1371/journal.pone.0348291)
Supplement: S1 File — (DOCX) [file pone.0348291.s001.docx]

**Claims and corresponding Community Notes and Expert Fact-Checks**

**False Claims**

1. Honestly, homelessness can easily be solved if people just got jobs or spent their money better.

***Community Note***

Finding and keeping a job whilst you’re homeless can be very difficult. There is a lack of access to clean clothes, keeping up personal hygiene, transportation matters and more. Employers may also be reluctant to hire people who have a criminal record or an inadequate educational level. Moreover people who are homeless may end up in that situation due to mental health issues, domestic violence or many other issues.

***Expert Fact-check***

Employment for people experiencing homelessness can be very difficult due to the lack of access to maintain facilities to maintain proper hygiene, clean clothes, transportation, and other wok necessities, contrary to the posts on social media claiming it is an easy solution.

1. There are lots of services for homelessness that people can turn to, like food banks, shelters and public bathrooms. I think there has been more than enough assistance provided to help people get off the streets.

**Community Note**

While there are many services available to assist people who are homeless, these are short term solutions. To solve the homelessness, it has been proposed that a permanent supportive housing model and minimising job insecurity are the approaches that need to be considered.

**Expert Fact-check**

Although there are a range of services readily available for people who are experiencing homelessness, these can only be viewed as short-term solutions. The long-term solution for homelessness has been proposed to be a compilation of coordinated supported services, affordable housing, and stable employment as opposed to the claims on social media that it can be dealt with by just seeking assistance from these services.

1. Most homeless people are on the streets, cold night tonight, hope they’re alright.

**Community Note**

The definition of ‘homelessness’ varies across the globe, but generally it involves lacking the elements of a home, per se. Many people who are homeless stay in dwellings or shelters.

**Expert Fact-Check**

According to official definitions, homelessness is not restricted to living on the streets. Contrary to many social media posts, many people who are homeless are residing in shared dwellings or shelters.

1. The government really needs to start providing more housing/accommodation for homeless people, if they want to try and fix this ever-growing problem. Otherwise, it just feels there’s no end to it. Really just sucks and feels like they don’t care.

**Community Note**

Simply providing housing/accommodation won’t be able to solve homelessness. This is the case as there is a growing gap between income and ability to pay living expenses. There is a widespread lack of affordable housing.

**Expert Fact-Check**

Affordable housing is only one factor contributing to homelessness, as there are a multitude of social and economic factors. Thus addressing homelessness is more than the provision of accommodation. Some social media posts claim that this is the means to solving homelessness despite there being other facts that that need to be addressed.

**True Claims**

1. Just heard that the rate of women and children becoming homeless is growing so fast. Domestic violence is a disease I swear.

**Community Note**

This is true. There is a common misconception that the majority of people who are homeless are men, however, in some countries like Australia and the United States, women make up half of the homelessness population. Domestic violence has been reported as one of the primary reasons.

**Expert Fact-check**

As a popular social post stated, half of the homeless population are women, as opposed to the misconception that there are more men than women. A considerable issue contributing to this is domestic violence.

1. The reality is anyone can become homeless. It doesn’t just selectively choose people with drug addictions or troublemakers. Literally anyone.

**Community Note**

Homelessness can indeed happen to anyone. There is no specific population group that will exclusively experience homelessness. There are several factors that can cause homelessness, such as domestic violence, mental health issues and alcohol abuse, unemployment, and more.

**Expert fact-check**

There is no demographic that will only be affected by homelessness. A range of factors can cause homelessness such as domestic violence, mental health issues, substance abuse and job insecurity.
